# The “Forgotten Disease” in a Healthy Young Adult: A Case Report of Lemierre’s Syndrome

**DOI:** 10.7759/cureus.93042

**Published:** 2025-09-23

**Authors:** Aimen Ayaz, Jishel Mathews, Salman Majeed, Arshad Jamil

**Affiliations:** 1 Respiratory Medicine, Sandwell and West Birmingham Hospitals NHS Trust, Birmingham, GBR; 2 Internal Medicine, Sandwell and West Birmingham Hospitals NHS Trust, Birmingham, GBR; 3 Internal Medicine, Northampton General Hospital NHS Trust, Northampton, GBR

**Keywords:** fusobacterium necrophorum, lemierre’s syndrome, sepsis, septic emboli, septic thrombophlebitis

## Abstract

Lemierre's syndrome is a rare, life-threatening condition caused by the anaerobic bacterium *Fusobacterium necrophorum, *typically affecting otherwise healthy individuals. The case report aims to raise awareness of this often-overlooked condition to help prevent associated morbidity and mortality. We present the case of a 22-year-old male who developed sepsis secondary to upper and lower respiratory tract infections. The patient experienced a four-week history of worsening cough and shortness of breath, followed by the onset of fever. His clinical condition rapidly deteriorated, leading to sepsis and type 1 respiratory failure, although invasive ventilation was not required. Computed tomography (CT) scans of the neck and chest revealed thrombosis of the right internal jugular and right pharyngeal veins, along with septic emboli in the lungs. Growth of *Fusobacterium necrophorum *in blood culturesconfirmed the diagnosis of Lemierre’s syndrome. Aggressive treatment with antibiotics and anticoagulants resulted in a full recovery and resolution of thrombosis. Although rare, Lemierre's syndrome remains a potentially fatal condition, and clinicians should maintain a high index of suspicion.

## Introduction

*Fusobacterium necrophorum*, a Gram-negative anaerobic bacillus commonly found in the oral cavity and oropharynx, is the primary etiological pathogen implicated in Lemierre’s syndrome. Following the advent of antibiotics, Lemierre’s syndrome became a rare condition and was subsequently termed the ‘forgotten disease’ [1,2].

Lemierre’s syndrome typically begins with an initial pharyngitis which spreads via the peritonsillar blood vessels to cause internal jugular vein (IJV) septic thrombophlebitis, followed by distant metastatic anaerobic septicaemia, most commonly affecting the lungs [3,4]. With a global incidence of approximately one in 1,000,000 and reported mortality rates ranging from 5-18%, Lemierre’s syndrome requires prompt diagnosis and management to reduce morbidity and long-term complications including cranial nerve palsies, functional impairments, and blindness [5-7]. The pathogenesis involves an increased bacterial affinity for blood vessels, leading to enhanced vascular and tissue permeability. This in turn activates the coagulation pathways, contributing to both the spread of infection and thrombus formation [6]. Differential diagnoses include Group A streptococcal pharyngitis and viral infections [7].

We present a case of Lemierre’s syndrome, highlighting its atypical presentation and complexities encountered during management.

This case was previously presented as a poster at the Association of Pakistani Physicians of Northern Europe (APPNE) Health Seminar, Walsall, United Kingdom, on September 28, 2024.

## Case presentation

A 22-year-old previously healthy male presented to the emergency department with a four-week history of an intermittent productive cough, along with a one-week history of fever, rigors, and chest pain. Physical examination revealed mild cervical lymphadenopathy, tachycardia, and dyspnoea. The remainder of the examination was unremarkable.

**Figure 1:**
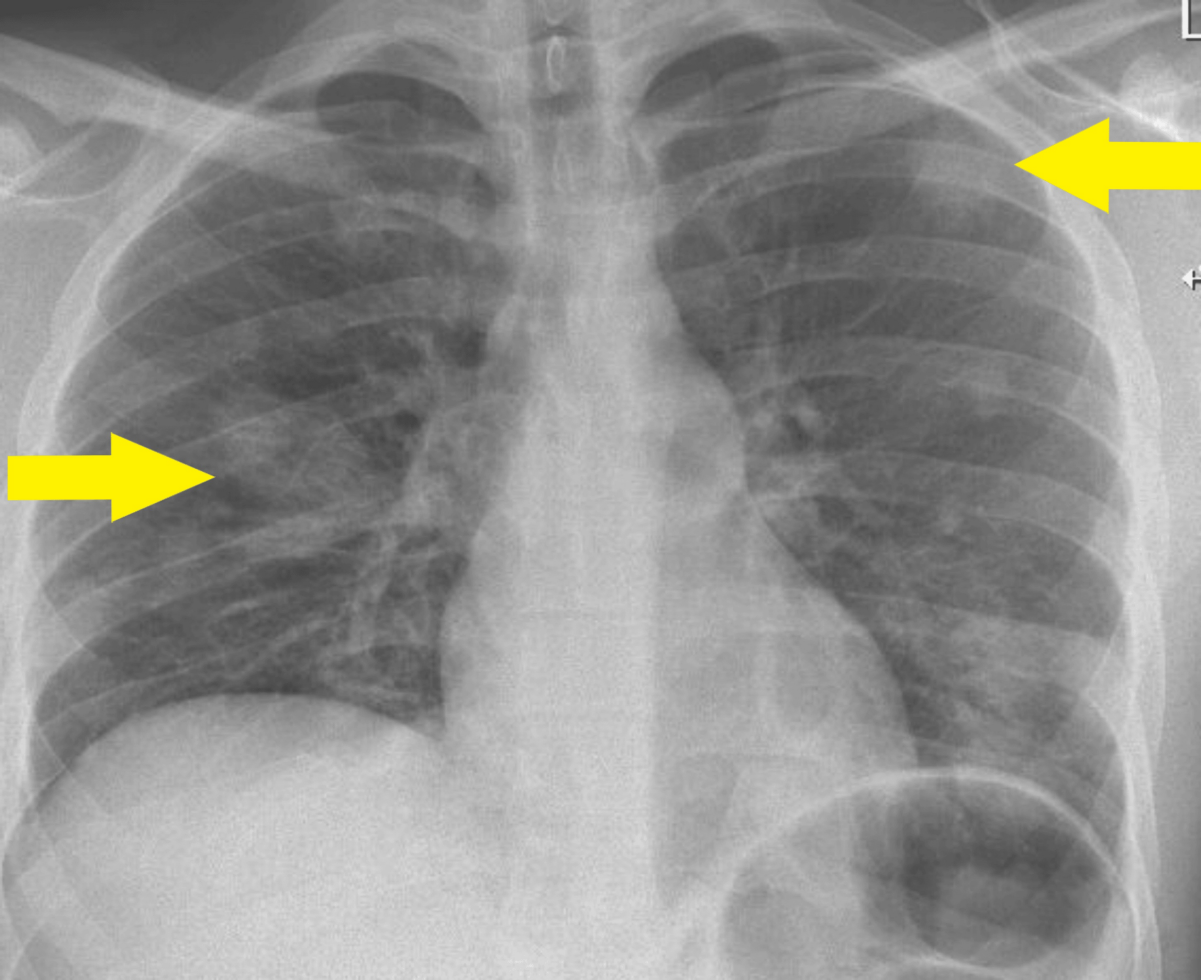
Posteroanterior (PA) chest X-ray on admission showing bilateral lung consolidations (yellow arrows)

Electrocardiography (ECG) showed sinus tachycardia. Initial blood tests indicated a severe infection, demonstrated by neutrophilia and elevated white blood cells (Table 1). Chest X-ray revealed bilateral pulmonary consolidations (Figure 1).

**Table 1:** Timeline of blood test results from initial hospital presentation to discharge

Laboratory tests	Day 1 Results	Day 2 Results	Day 4 Results	Day 5 Results	Day 10 Results	Day 15 Results	Reference range
White blood cell (×10⁹/ L)	31.40	33.60	24.80	26.80	19.60	13.20	(4-11)
Red blood cell (×10⁹/ L)	4.67	4.04	3.24	3.10	2.97	2.94	(4.5-6.5)
Neutrophils (×10⁹/ L)	27.48	29.64	20.0	22.65	14.57	9.60	(2-7.5)
Lymphocytes (×10⁹/ L)	1.48	2.42	3.32	2.59	2.60	2.52	(1.5-4.5)
Monocytes (×10⁹/ L)	2.42	1.48	1.07	1.26	1.38	0.88	(0.2-0.8)
Basophil (× 10⁹/ L)	0.03	0.03	0.10	0.11	0.06	0.07	(0-0.1)
Eosinophil (×10⁹/ L)	0.00	0.03	0.05	0.11	0.09	0.13	(0-0.4)
Hemoglobin (g/L)	138	117	94	91	88	85	(130-180)
Hematocrit (L/L)	0.393	0.330	0.263	0.257	0.249	0.253	(0.4-0.52)
Platelets (×10⁹/ L)	71	90	198	223	542	498	(150-450)
Sodium (mmol/L)	131	129	133	134	131	132	(133-146)
Potassium (mmol/L)	4.2	3.8	3.8	4.1	4.2	4.4	(3.5-5.3)
Urea (mmol/L)	7.4	5.9	3.5	3.4	4.0	3.6	(2.5-7.8)
Creatinine (µmol/L)	115	80	85	78	62	59	(64-111)
Estimated glomerular filtration rate (mL/min/1.73m²)	78	>90	>90	>90	>90	>90	(>90)
C-reactive protein (mg/L)	383	346	218	189	148	74	(0-5)
Bilirubin (µmol/L)	59	32	38	28	16	9	(5-26)
Alanine aminotransferase (U/L)	81	52	32	36	25	20	(0-55)
Alkaline phosphatase (U/L)	224	185	126	129	81	66	(30-130)

**Figure 2:**
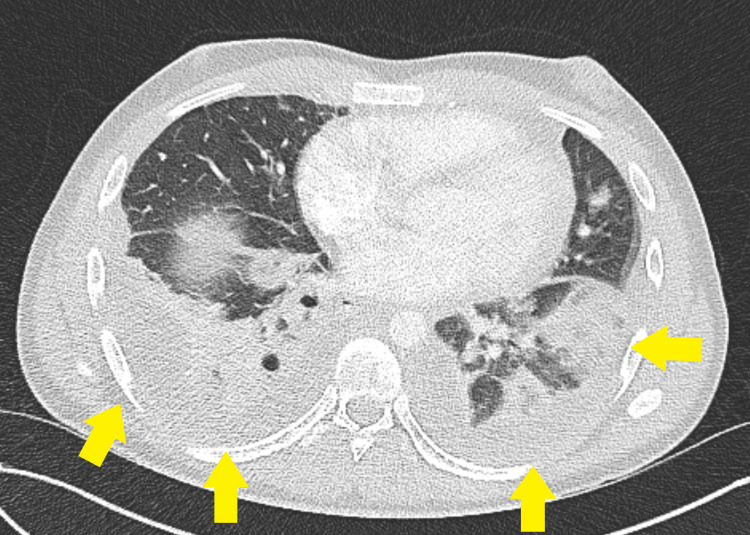
Axial contrast-enhanced computed tomography pulmonary angiography (CTPA) of the chest demonstrating extensive bilateral patchy areas of consolidations, pulmonary infarcts, and small bilateral pleural effusions (yellow arrows)

**Figure 3:**
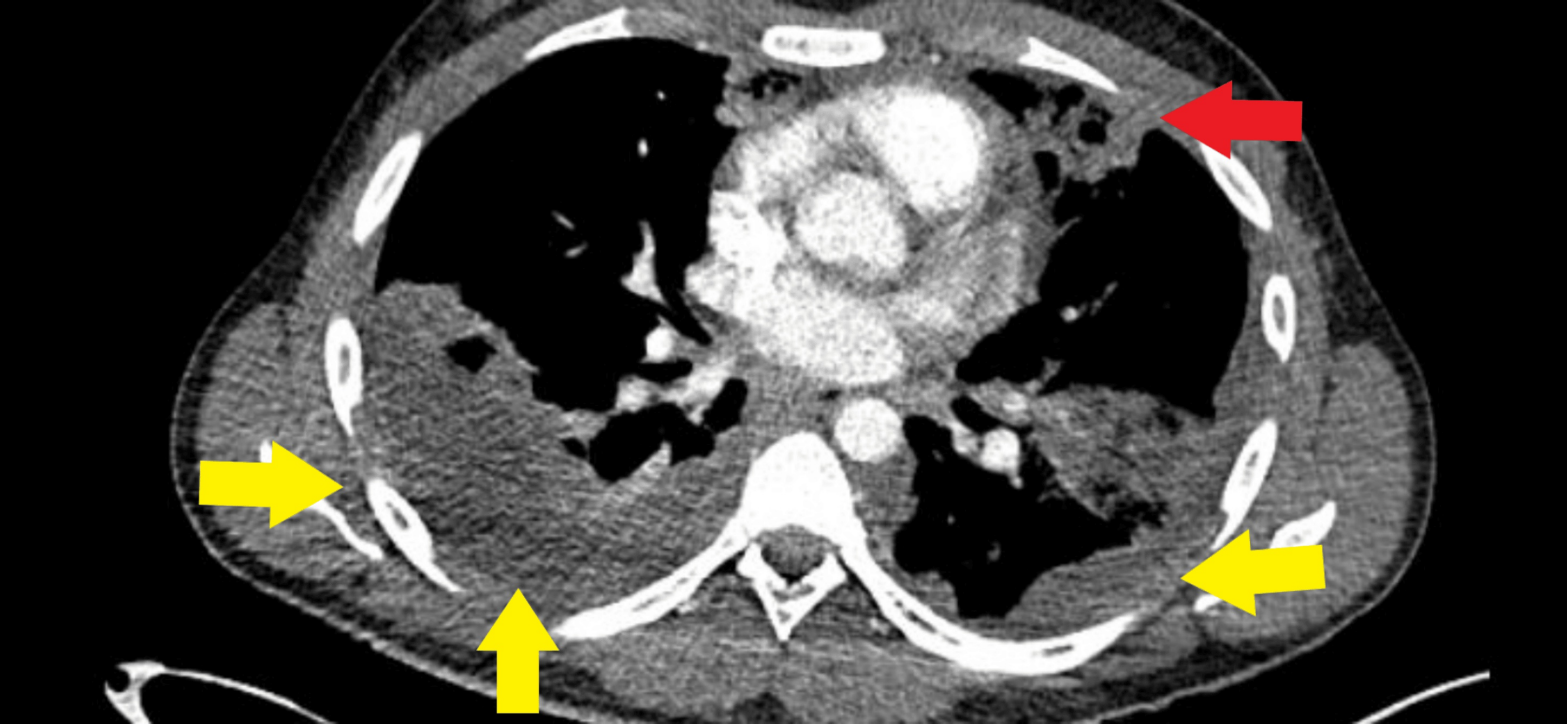
Axial contrast-enhanced computed tomography pulmonary angiography (CTPA) of the chest demonstrating a patchy area of cavitated consolidation (red arrow) and small bilateral pleural effusions (yellow arrows)

Based on a working diagnosis of atypical pneumonia, clarithromycin and intravenous piperacillin-tazobactam were initiated. Axial contrast-enhanced computed tomography pulmonary angiography (CTPA) of the chest showed bilateral cavitated consolidations with features suggestive of septic emboli and associated pulmonary infarcts (Figure 2 and Figure 3). A bedside transthoracic echocardiogram revealed no evidence of cardiac vegetations. 

On day two after admission, blood cultures revealed growth of Gram-negative bacilli. The patient developed increased oxygen requirement (up to 4 L/min) and persistent fever >38°C. A stat dose of intravenous gentamicin was administered; however, the initial antibiotic regimen was continued.

**Figure 4:**
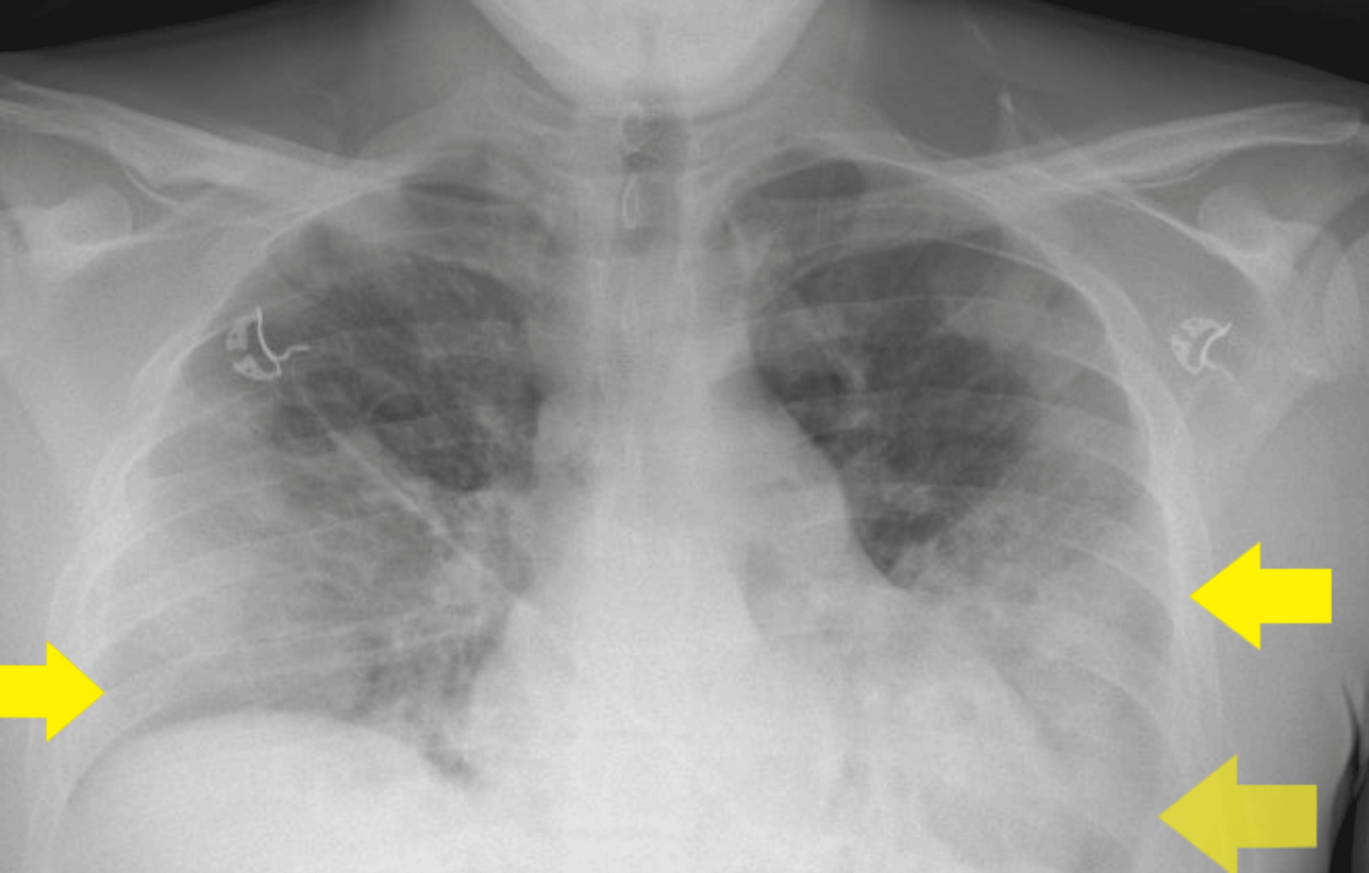
Anteroposterior (AP) chest X-ray on day four after admission showing confluent consolidation in both mid and lower lung zones and bilateral pleural effusions, consistent with worsening infection (yellow arrows)

**Figure 5:**
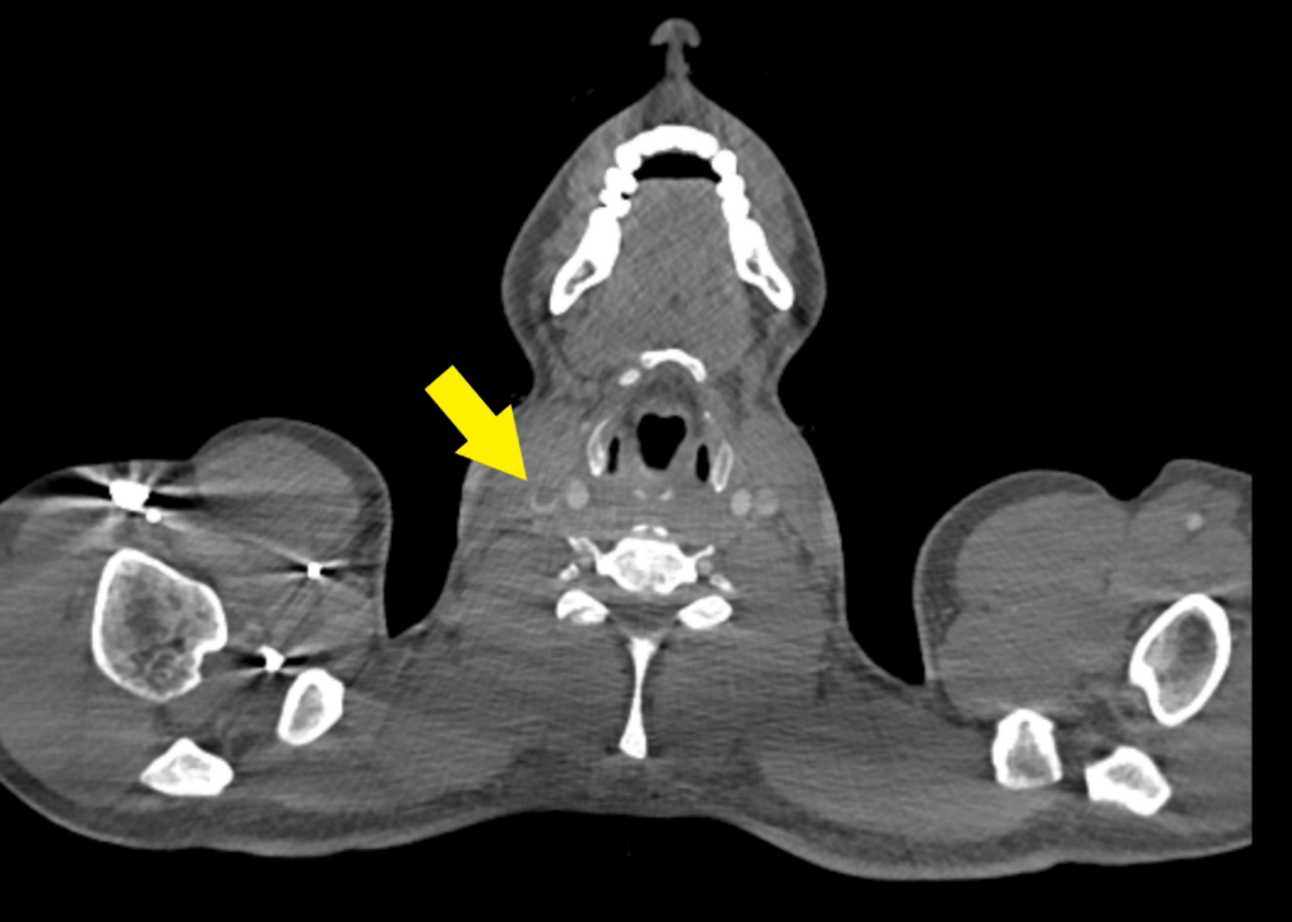
Axial contrast-enhanced computed tomography (CT) of the neck showing thrombus in the right internal jugular vein (IJV) (yellow arrow)

On day four after admission, blood cultures grew *Fusobacterium necrophorum*. Intravenous metronidazole was initiated alongside piperacillin-tazobactam, and oral clarithromycin was discontinued. A repeat chest X-ray revealed worsening bilateral consolidations (Figure 4). An urgent contrast-enhanced CT (CECT) of the neck was performed to assess for occult abscesses. The CECT scan of the neck confirmed the presence of a thrombus in the right IJV and right pharyngeal veins; however, no parapharyngeal abscess or thrombosis of the skull veins was identified (Figure 5). Therefore, the patient was started on a therapeutic enoxaparin (1.5 mg/kg subcutaneously once daily) for IJV thrombosis. The haematology team recommended continuing low molecular weight heparin (LMWH) for at least three months. 

**Figure 6:**
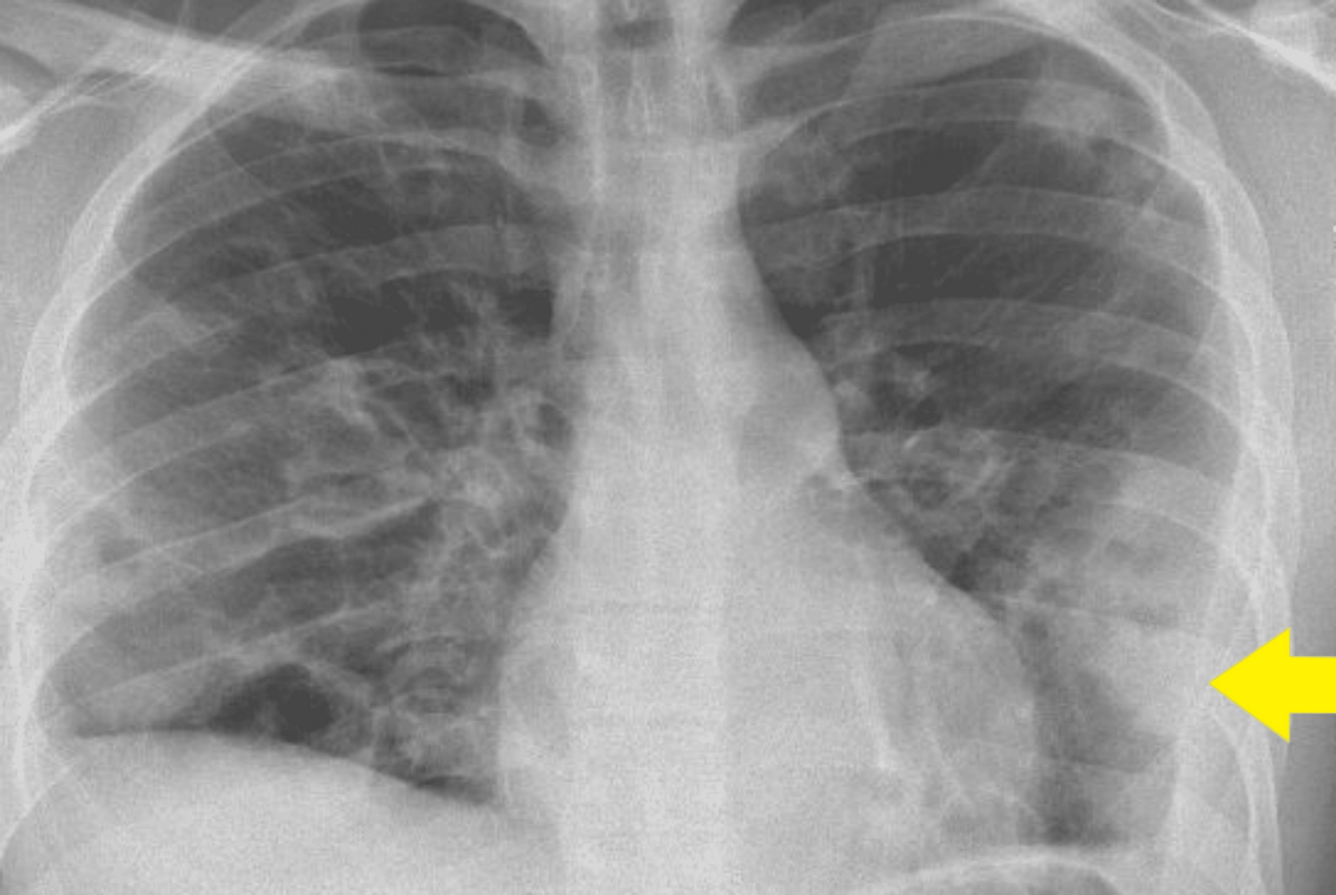
Posteroanterior (PA) chest X-ray on day 14 of admission showing patchy areas of consolidations, consistent with resolving chest infection (yellow arrow)

**Figure 7:**
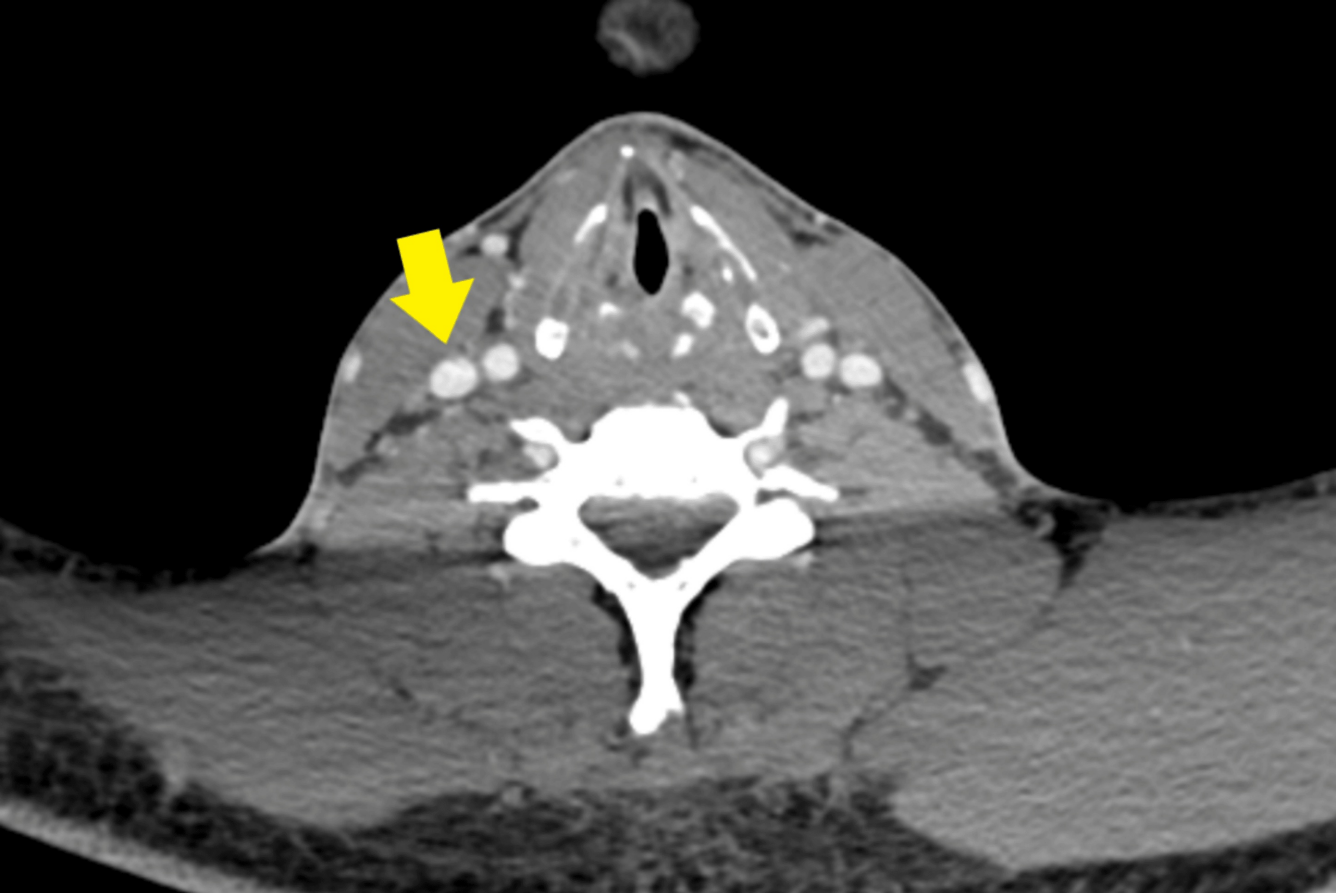
Axial contrast-enhanced computed tomography (CT) of the neck showing complete resolution of thrombus in the right internal jugular vein (IJV) (yellow arrow)

The patient demonstrated improvement in inflammatory markers and decreased oxygen requirements. A repeat chest X-ray on day 14 of admission showed significant resolution of pulmonary consolidations (Figure 6). Haemodilution was considered as the cause of anaemia, as the patient received intravenous fluids while unwell. Reactive thrombocytosis was attributed to sepsis. There was no evidence of haemolysis or bleeding at any point during admission (Table 1). Intravenous antibiotics were switched to oral amoxicillin and metronidazole on day 14 of admission. The infectious diseases team recommended a minimum of four weeks of antibiotic therapy. The patient was discharged after a two-week hospital stay, with follow-up arranged for anticoagulation review, a three-month follow-up CECT of the neck to assess resolution. A follow-up appointment with the respiratory virtual clinic was also arranged. Follow-up CECT of the neck showed complete resolution of right IJV thrombosis, with no residual filling defects (Figure 7).

## Discussion

Lemierre’s syndrome, also known as post-anginal sepsis, was first described in the early 1900s. Andre Lemierre reported 20 cases of anaerobic septicemias originating from simple tonsillitis and progressing to thrombophlebitis of the IJV, with a reported mortality rate of 90% [8,9]. In approximately 33% of cases, the first clinical sign is pharyngitis or a sore throat; however, it may also present as neck mass, neck pain, or even limb weakness [7].

This case report highlights an atypical presentation of Lemierre’s syndrome, in which life-threatening bacterial septicemia likely originated from pharyngitis without overt oropharyngeal findings. The mild cervical lymphadenopathy noted on examination may have been misinterpreted as neck swelling or tenderness associated with IJV thrombophlebitis [9,10].

Microbiological confirmation of *Fusobacterium necrophorum* in blood cultures supported the diagnosis and prompted a CT of the neck, which confirmed an IJV thrombus. Additional imaging modalities for diagnosing Lemierre’s syndrome include MR venography, which has the highest sensitivity for detecting IJV thrombus and Doppler ultrasound, which is more cost-effective but less sensitive [5,11].

Prior to the introduction of antibiotics, Lemierre’s syndrome carried a high mortality rate. Historical management involved supportive care or surgical ligation of the IJV to prevent the spread of septic emboli [8,11]. Current treatment focuses on early administration of intravenous antibiotics to ensure adequate penetration into the fibrin thrombus [2].

In this case, the patient was treated with intravenous piperacillin-tazobactam and metronidazole. As approximately 25% of *F. necrophorum* strains produce beta-lactamase, it is imperative to use beta-lactamase-resistant antibiotics, such as piperacillin-tazobactam [12]. In refractory cases unresponsive to medical management, several case reports have described the successful use of surgical interventions. These include ligation of the IJV, incision and drainage of pharyngeal abscesses, tooth extraction and craniotomy [13,14].

The use of anticoagulation in Lemierre’s syndrome remains controversial. Due to the low incidence of the condition and lack of randomized control trials, treatment decisions are made on a case-by-case basis. A case study from Sweden reported no statistically significant difference in clot resolution between patients who received anticoagulation and those who did not [15]. One hypothesis is that the fibrin clots may sequester bacteria, and anticoagulants may enhance greater antibiotic penetration by breaking down the thrombus [16].

In this case, local haematology team recommended initiating anticoagulation due to clinical deterioration despite 48 hours of intravenous antibiotics. The patient made a full recovery, with no evidence of post-treatment complications or neurological sequelae at the three-month follow-up after discharge.

## Conclusions

This case report highlights that although Lemierre’s syndrome is rare, it can be life-threatening. It often presents a diagnostic challenge due to the high prevalence of benign oropharyngeal infections, which may lead to delayed diagnosis and an increased risk of complications. The rapid progression from a benign sore throat to severe sepsis, septic emboli in otherwise healthy young individuals highlights the importance of early recognition and prompt initiation of appropriate treatment, primarily with antibiotics. A multi-disciplinary approach is essential to ensure effective patient care. Physicians must remain vigilant for Lemierre’s syndrome, particularly in atypical presentations, given its diagnostic and therapeutic complexities. 
